# Stress, social support, and racial differences: Dominant drivers of exclusive breastfeeding

**DOI:** 10.1111/mcn.13459

**Published:** 2022-11-21

**Authors:** Chinwoke Isiguzo, Dara D. Mendez, Jill R. Demirci, Ada Youk, Gabriella Mendez, Esa M. Davis, Patricia Documet

**Affiliations:** ^1^ Behavioral and Community Health Sciences University of Pittsburgh Graduate School of Public Health Pittsburgh Pennsylvania USA; ^2^ Department of Epidemiology University of Pittsburgh Graduate School of Public Health Pittsburgh Pennsylvania USA; ^3^ Department of Health Promotion and Development, School of Nursing University of Pittsburgh Pittsburgh Pennsylvania USA; ^4^ Department of Biostatistics, Graduate School of Public Health University of Pittsburgh Pittsburgh Pennsylvania USA; ^5^ Orthopedic Foot and Ankle Center Worthington Ohio USA; ^6^ Department of Medicine, School of Medicine University of Pittsburgh Pittsburgh Pennsylvania USA

**Keywords:** breastfeeding, Ecological Momentary Assessment, exclusive breastfeeding, perceived social support, perceived stress, racial disparity, received social support

## Abstract

Exclusive breastfeeding is recommended for 6 months; however, many childbearing people wean their infants before 6 months. Psychosocial factors such as stress, social support and race are significant determinants of breastfeeding; however, few studies have longitudinally explored the effect of perceived stress and various forms of social support on exclusive breastfeeding. We used quantitative methodologies to examine exclusive breastfeeding, perceived stress and social support among 251 participants from the Postpartum Mothers Mobile Study. Participants between 18 and 44 years were recruited during pregnancy (irrespective of parity) and completed surveys in real‐time via Ecological Momentary Assessment up to 12 months postpartum from December 2017 to August 2021. We measured perceived stress with the adapted Perceived Stress Scale and perceived social support with the Multi‐dimensional Social Support Scale. Received social support was measured using a single question on breastfeeding support. We conducted a mixed‐effects logistic regression to determine the effect of stress, race and social support on exclusive breastfeeding over 6 months. We examined the moderation effect of perceived social support and breastfeeding support in the relationship between perceived stress and exclusive breastfeeding. Black, compared with White, participants were less likely to breastfeed exclusively for 6 months. Participants who reported higher perceived stress were less likely to breastfeed exclusively for 6 months. Perceived social support moderated the relationship between perceived stress and exclusive breastfeeding (odds ratio: 0.01, 95% confidence interval: 0.001–0.072). However, breastfeeding support directly increased the likelihood of exclusive breastfeeding over 6 months. Perceived stress is negatively associated with exclusive breastfeeding. Birthing people who intend to breastfeed may benefit from perinatal support programs that include components to buffer stress.

## Key messages

- Childbearing people who reported higher perceived stress were less likely to breastfeed exclusively.
- Perceived social support moderated perceived stress, thereby increasing the likelihood of exclusive breastfeeding.
- Received social support did not moderate perceived stress; however, it directly increased the likelihood of exclusive breastfeeding.
- Overall, Black childbearing people in our study were less likely to exclusively breastfeed.

## 1 BACKGROUND

Breastfeeding is one of the most significant, cost‐effective public health interventions to optimize maternal and child health (Jones et al., 2003; World Health Organization [WHO], 2005). Exclusive breastfeeding for the first 6 months of life is recommended by the WHO and the American Academy of Pediatrics, owing to the dose‐dependent health and developmental effects of breastfeeding for parents and child (Centers for Disease Control and Prevention [CDC], 2020; Raisler et al., 1999; WHO, 2001). However, most childbearing people in the United States stop breastfeeding or introduce infant formula or complementary foods before 6 months (CDC, 2020).

From the 2018–2019 National Immunization Survey of infants born in 2018 in the United States, 83.9% were ever breastfed (CDC, 2020). This survey also reported that the percentage of infants still breastfeeding at 6 months was 56.7% and 35.0% at 12 months (CDC, 2020). Only 46.3% of children were exclusively breastfed through 3 months, which reduced to 25.8% at 6 months (CDC, 2020). Comparing infants born in 2018 with those born in 2011, exclusive breastfeeding rates at 3 and 6 months have risen slowly and steadily by 5.6% and 6.6%, (CDC, 2020) likely due to increased breastfeeding promotion efforts (Bibbins‐Domingo et al., 2016). However, this increase is not equitably distributed across the United States (Anstey et al., 2017; Geraghty et al., 2012). Among Black infants, 39.3% were exclusively breastfed at 3 months and 19.8% at 6 months, whereas the rates were considerably higher among White infants (i.e., 50.6% for 3 months and 28.8% at 6 months) (CDC, 2020).

Breastfeeding practices are influenced by historical, socioeconomic, cultural, physiological, and psychosocial factors (Rollins et al., 2016; Shiraishi et al., 2020). Sociodemographic factors and maternal characteristics such as age, race/ethnicity, education, employment, income, marital status, perceptions of insufficient milk supply, beliefs, knowledge, attitudes about breastfeeding, and parity have been widely reported as important factors associated with breastfeeding (Bentley et al., 2003; Johnson et al., 2015a; Meyerink & Marquis, 2002). Structural barriers such as suboptimal maternity care practices in birth facilities, lack of workplace breastfeeding support, and predatory advertising from infant formula manufacturers can also influence breastfeeding practices and duration (Johnson et al., 2015a; Rosenberg et al., 2008). In addition, physiological barriers such as unresolved pain can lead to breastfeeding discontinuation (Odom et al., 2013; Shiraishi et al., 2020).

Researchers have also shown that maternal psychosocial factors such as stress and social support are major determinants of breastfeeding (de Jager, Skouteris, et al., 2013; de Jager, Broadbent, et al., 2014; Islam et al., 2017). Maternal stress is associated with adverse perinatal outcomes, including breastfeeding (Ahluwalia et al., 2001; Dole et al., 2003; Mezzacappa, 2004). The relationship between stress and breastfeeding appears complex, and the directionality is unclear (Islam et al., 2021; Nagel et al., 2021; Thome et al., 2006). Experimental studies demonstrate that maternal stress seems to interfere with the release of oxytocin, the hormone responsible for the milk ejection reflex, and if prolonged, can lead to reduced breastmilk production (Dewey, 2001). When going well, breastfeeding is also associated with decreased stress and improved mood among lactating parents (Mezzacappa et al., 2000). This beneficial effect of breastfeeding is said to be mediated by oxytocin and prolactin, hormones necessary for breastfeeding, and is linked to reducing depression and stress (Arletti & Bertolini, 1987; Uvnas‐Moberg & Petersson, 2005).

Exposure to stress is common to all; however, in the United States, Black populations are more susceptible to chronic stress, including perinatal stress, compared with White people, which stems from racial discrimination (Dole et al., 2004; Stancil et al., 2000). Chronic stressors activate the hypothalamic–pituitary–adrenal axis and sympathetic, immune and cardiovascular systems, releasing higher levels of stress hormones such as pro‐inflammatory cytokines and cortisol in the effort to restore allostasis (Hawkley et al., 2007; Premji, 2014; Tull et al., 2005). Repeated allostatic responses activated during those stressful situations cause ‘wear and tear’ to the body and inadvertently lead to allostatic overload (Premji, 2014). Black populations in the United States have been shown to have a higher allostatic load due to racism, poverty, and perceived stress (Wallace & Harville, 2013). High allostatic load has been posited to contribute to worse health outcomes, including adverse birth outcomes and health risk behaviors such as substance use (Giscombé & Lobel, 2005; Wallace & Harville, 2013).

Social support is one of the essential resources for navigating stressors that can increase substantially postpartum (Islam et al., 2021). Researchers have demonstrated that social support, including from family, friends, peers, and health professionals, is critical to breastfeeding establishment and continuation (Raj & Plichta, 1998). Social support depends on the availability and quality of social relationships, which may moderate stress exposures (Cassel, 1976; Cobb, 1976). This implies that the effect of stress may be more significant among those who lack social ties compared with those who have supportive relationships with others. In a study conducted in Bangladesh, maternal stress negatively influenced exclusive breastfeeding, whereas social support buffered the relationship (Islam et al., 2021). Other studies have found no significant relationship between stress, social support and exclusive breastfeeding (Akman et al., 2008; Jalal et al., 2017).

Researchers have described the relationship between social support and well‐being using either the main‐effect or buffering models (Cohen & Wills, 1985). The main‐effect model suggests that social support produces direct and beneficial effects on well‐being, independent of stressors. Alternatively, the buffering model asserts that social support protects individuals by mitigating the adverse effects of stressors (Cohen & Wills, 1985). This has led to categorizing social support into two major types: perceived and received social support (Berkman & Syme, 1979; Helgeson, 1993; Vangelisti, 2009). Perceived social support assesses the extent to which people believe support is available to them, whereas received support assesses specific supportive acts that have occurred (Helgeson, 1993).

Previously, researchers have examined how the two types of social support might impact health. Several studies posit that the perception of support is associated with reduced mortality and seems to be a better predictor of health outcomes than the actual receipt of support (Cohen & Hoberman, 1983; Cohen & Wills, 1985; Eagle et al., 2018; Hartley & Coffee, 2019; Wethington & Kessler, 1986). In contrast, other studies have shown that received support has a more significant effect than perceived support following a stressful event, especially if a single stressor is examined and the support is specific to the stressor (Dunkel‐schetter & Bennett, 1990). The conflicting evidence makes health intervention challenging. The distinction between perceived and received social support in the relationship between stress and exclusive breastfeeding is limited.

To date, studies examining the relationship between stress and exclusive breastfeeding have been cross‐sectional (Dozier et al., 2012; Dugat et al., 2019) and, as such, the temporal association between stress and breastfeeding patterns cannot be established. Some of these studies had insufficient statistical power (Mezzacappa, 2004) or measured stressful life events (i.e., financial, emotional, traumatic, and partner‐associated) but not perceived stress (Dozier et al., 2012; Dugat et al., 2019). Others have focused on a particular group, such as low‐income childbearing people (Dozier et al., 2012). Notably, measuring individual stress through stressful life events is complex because of the lack of consistency in its definition (Epel et al., 2018). In addition, no published study has demonstrated the role of perceived or received social support in the relationship between perceived stress and exclusive breastfeeding using temporal methods, such as the Ecological Momentary Assessment (EMA).

Our objective, therefore, is to examine the relationship between perceived stress and exclusive breastfeeding, and the moderating effects of perceived and received social support on this relationship. In addition, we examined the racial differences in exclusive breastfeeding.

## 2 METHODS

### 2.1 Study design

Postpartum Mothers Mobile Study (PMOMS) is a longitudinal study designed to understand the contextual, behavioral, psychosocial, and clinical factors related to racial disparities in postpartum weight and cardiometabolic health (Davis et al., 2021; Mendez et al., 2019, 2020). It is an ancillary study to the Comparison of Two Screening Strategies for Gestational Diabetes (GDM‐2) (Abebe et al., 2017), a randomized controlled trial conducted in a single birthing hospital in Southwestern Pennsylvania.

The GDM‐2 study began recruitment in 2015 and required two study visits. Starting in December 2017, participants were approached at these visits, screened and enrolled in PMOMS (Davis et al., 2021; Mendez et al., 2019, 2020). PMOMS recruited 284 participants aged 18–44 years from the GDM‐2 clinical trial and directly recruited an additional 29 participants. The study participants were recruited between 18 and 28 weeks of gestation and followed up to 1 year postpartum. Participants completed EMA surveys assessing physical activities, diet, breastfeeding behaviors, stress, and other psychosocial factors from 18 to 36 weeks of pregnancy through 12 months postpartum (Mendez et al., 2019). Data collection for the PMOMS ended in August 2021.

### 2.2 Data collection

EMA via mobile device/smartphone was the primary data collection method for PMOMS (Mendez et al., 2019). EMA data collection occurs in real‐time and participants complete surveys capturing momentary states, behavior, and conditions multiple times or as repeated measures (Lazarides et al., 2020, Shiffman et al., 2008). EMA as a data collection method minimizes recall bias, which is common in most retrospective studies (Shiffman et al., 2008).

Participants in PMOMS received text message prompts and completed daily EMA surveys via a mobile phone app at the beginning of day (BOD), end of day (EOD), and random times throughout the day. Participants were allowed to select when to complete the BOD and the EOD surveys with at least 9 hours between these two surveys. Participants received random EMA surveys 0–3 times per day between the BOD and EOD survey times, targeting a mean of 1 random assessment per day over a 7‐day period beginning at recruitment (18–36 weeks of pregnancy) through 12 months postpartum (Mendez et al., 2019). Demographic data were collected at baseline (after being enrolled) in the GDM‐2 study. Breastfeeding questions were asked at the EOD. Questions on perceived stress and received social support (breastfeeding support) were asked as part of the random EMA surveys. Questions on perceived social support (Multidimensional Scale of Perceived Social Support [MSPSS]) were asked as part of the non‐EMA surveys during the exit survey at 12 months. A detailed description of the data collection, including the study protocol has been described elsewhere (Mendez et al., 2019).

## 3 MEASURES

### 3.1 Outcome variable

#### 3.1.1 Exclusive breastfeeding: 24 h recall

Breastfeeding data were collected via the EOD survey. For every 42‐day block to 6 months postpartum, breastfeeding questions were prompted 8 weekend days and 20 weekdays. To determine whether a participant exclusively breastfed, the following question was asked ‘Are you exclusively breastfeeding?’ with answer options of ‘yes’ or ‘no’. Participants selected ‘yes’ if they breastfed exclusively that day or selected ‘no’ if they did not breastfeed exclusively that day. The WHO definition of exclusive breastfeeding (‘exclusive BF includes only breastmilk [including expressed/pumped milk] with the exception of medicine, vitamins or oral rehydration’) was included in the question to ensure that participants understood the operational definition. Participants continue to receive surveys asking about exclusive breastfeeding irrespective of their previous day's response.

### 3.2 Independent variables

#### 3.2.1 Perceived stress

The random EMA measure of stress was adapted from the Perceived Stress Scale (PSS) 10 and 4 (Cohen, Kamarck et al., 1983; Cohen, 1988); we used three items from the Cohen's PSS4 and one item from PSS10. Cronbach *α* for PSS10 and PSS4 are 0.78 and 0.60, respectively (Lee, 2012). PSS 4 and 10 have been used among Black postpartum people in the United States and validated among perinatal populations (Karam et al., 2012; Thibeau et al., 2016). Random EMA prompts to assess perceived stress with the adapted PSS instrument were delivered 0–3 times per day, targeting a mean of one random assessment per day over a 7‐day period between 18 and 36 weeks pregnancy and 12 months postpartum (Mendez et al., 2019). We used the PSS as a continuous variable to determine the degree to which participants appraise situations in their lives as stressful, uncontrollable, unpredictable and difficult. The items were scored on a 5‐point Likert scale, with responses ranging from never (0) to a lot (4).

### 3.3 Moderating variables

#### 3.3.1 Perceived social support

Perceived social support was measured using the MSPSS (Zimet et al., 1988). The MSPSS was measured at one point in time as a non‐EMA measure at 12 months postpartum. MSPSS has three subscales that measure an individual's perception of support from three sources: family, friends, and significant other. The widely validated MSPSS scale has 12 items and a Cronbach's *α* of 0.88; however, we used 11 items to reduce survey burden on the respondents. On a 4‐point Likert scale, the respondents indicated the extent to which each statement described their current relationships with their friends, family and significant other. Responses ranged from 0 (*Strongly disagree*) to 4 (*Strongly agree*). We recoded MSPSS as a dichotomous variable, grouped as: no (0–2 points) and yes (3 and 4 points).

#### 3.3.2 Received social support (breastfeeding support)

To measure received social support specifically for breastfeeding, participants completed a single investigator‐created item measuring received breastfeeding support. Participants received random prompts in the day asking them about the breastfeeding support they received while breastfeeding. The question was, ‘Is there a person/group/organization (e.g., family, professionals) that is helping you or providing any support (e.g., resources, emotional) to continue to breastfeed?’ Responses were either ‘Yes’ or ‘No’.

#### 3.3.3 Demographic characteristics

Self‐reported demographic characteristics were collected at baseline (18–36 weeks of pregnancy) and included maternal age, race (Black or White), educational attainment (high school diploma/GED or less and some college degree and above), employment (employed and unemployed), income (<$30,000 or $31,000 and above). Participants identified as Asian, Native Hawaiian/Other Pacific Islander, White, Black/African American, Multi‐racial and other race. We excluded racial categories other than White or Black for this analysis due to small sample size. Black participants, regardless of their ethnicity, were included in the analysis.

### 3.4 Analytic sample

The overall PMOMS population included 313 participants. Of these, 284 answered EMA survey questions on breastfeeding. As our aim was to compare psychosocial factors that influence exclusive breastfeeding between White and Black participants, we excluded participants that identified as Asian, Native Hawaiian or multiracial and others; this resulted in 78 Black and 173 White participants, and a total of 251 participants in the final analytic sample.

### 3.5 Statistical analyses

We calculated the internal consistency of the adapted Cohen Perceived scale and the MSPSS using Cronbach's *α*. We conducted a descriptive analysis of the demographic characteristics of participants and generated individual panel plots for repeated measures of exclusive breastfeeding and perceived stress. We also tested for multicollinearity among the sociodemographic factors and the independent variables. All independent and sociodemographic variables were included since their variance inflation factors were <5.

To examine the relationship between exclusive breastfeeding (a dichotomous variable), perceived stress and social support, we conducted a mixed‐effects logistic regression model with random intercepts and unstructured covariance matrices to address clustering of individual responses (panel‐data regression). This approach allowed each subject to deviate from the overall mean response by a person‐specific constant that applies equally over time. First, we identified covariates included in the final model by conducting a bivariate test of association between exclusive breastfeeding and sociodemographic factors, and independent variables. Next, we specified a null and two full models with random intercepts.

We examined the moderation effect of the two forms of social support (i.e., multidimensional scale of social support and breastfeeding social support) considered perceived and received support, respectively, on perceived stress. In addition, we also examined the moderation effect of race on perceived stress. Model 1 included an interaction term between perceived social support and perceived stress, whereas Model 2 included an interaction term between received social support and perceived stress. We graphically illustrated the interaction between the two forms of social support and perceived stress using the marginplot command in STATA. We used log‐likelihood, Bayesian Information Criterion and Akaike's Information Criterion to ascertain model goodness‐of‐fit. In addition, we calculated the Proportional Change in Variance to estimate the total variance attributable to the independent variables in the models. STATA/S.E 16.0 was used to conduct all statistical analyses (StataCorp LP, 2019).

## 4 RESULTS

### 4.1 Sociodemographic factors

Table 1 shows the characteristics of the respondents in the study stratified by race. Over 67% of the participants were White and 32% Black. More than half of the Black participants were unemployed, whereas about a third of White participants were unemployed. More than 90% of White participants had a college degree, whereas about 51% of Black participants had a college degree. White participants also had higher incomes than Black participants.

**Table 1:** Characteristics of the respondents stratified by race (White and Black)

Sociodemographic variables	All (*N* = 251)	Mean (SD)/%	White (*n* = 173)	Mean (SD)/%	Black (*n* = 78)	Mean (SD)%
**Maternal age in years, M (SD)**	251	29.9 (4.9)	173	31.0*(4.5)	78	27.5 (4.6)
**Employment status %**						
Employed	175	69.7	137	79.2	38	48.7
Unemployed	76	30.3	36	20.8	40	51.3
**Marital status %**						
Unmarried	128	42.5	45	26.0	14	18.0
Married or partnered	173	57.5	128	74.0	64	82.0
**Income level** %						
<$30,000	96	38.3	32	18.5	64	82.1
$31000 and above	155	61.8	141	81.5	14	17.9
**Education level** %						
High school diploma/GED or less	55	21.9	17	9.8	38	48.7
Some college degree or higher	196	78.1	156	90.2	40	51.3

### 4.2 Reliability of perceived stress and perceived social support measures

The adapted Cohen's Perceived Stress and the Multidimensional Scale of Social Support showed high internal consistency of Cronbach's *α* 0.81 and 0.95, respectively.

### 4.3 Factors associated with exclusive breastfeeding

Table 2 shows bivariate analyses of psychosocial and maternal characteristics associated with the odds of exclusive breastfeeding. In this unadjusted model, among those with higher levels of stress there was a lower likelihood of exclusive breastfeeding (odds ratio [OR]: 0.92, 95% confidence interval [CI]: 0.88–0.95). Overall, compared with White participants, Black participants were less likely to exclusively breastfeed over 6 months. Participants who received social support (breastfeeding social support) and who perceived that they had social support (perceived social support) were two and four times as likely to exclusively breastfeed, respectively. Participants with a college education, high income, employed and older were more likely to breastfeed exclusively. Table 3 shows an adjusted mixed‐effect model examining the relationship between exclusive breastfeeding and maternal characteristics. In this adjusted model, perceived stress and race were the only factors associated with exclusive breastfeeding. Participants who reported higher levels of stress were less likely to exclusively breastfeed.

**Table 2:** Unadjusted model examining each maternal psychosocial factors and characteristics in relation to repeated reports of exclusive breastfeeding

	Unadjusted model	
Fixed effect	Odds ratio (95% CI)	*p*
Perceived stress^a^	0.92 (0.88–0.95)	<0.001
Received support^b^ (Breastfeeding support)	2.09 (1.63–2.67)	<0.001
Perceived social support^c^	4.42 (1.01–19.30)	0.047
Race		
White	Ref	Ref
Black	0.01 (0.002–0.023)	<0.001
Education		
High school diploma/GED or less	Ref	Ref
College degree or higher	96.25 (25.61–361.66)	<0.001
Income		
<$30,000	Ref	Ref
$31,000 and above	315.28 (105.26–944.36)	<0.001
Employment status		
Employed	Ref	
Unemployed	0.02 (0.01– 0.07)	<0.001
Maternal age	1.28 (1.12–1.46)	<0.001

Abbreviation: CI, confidence interval.

^a^ Perceived Stress: Adapted Cohen's Perceived Stress Scale. Operationalized as a continuous variable.

^b^ Received support (Breastfeeding support): Is there a person/group/organization (e.g., family, professionals) that is helping you or providing any support (e.g., resources, emotional) to continue to breastfeed? Response option is ‘Yes’ or ‘No’.

^c^ Perceived social support: Adapted Multidimensional Scale of Perceived Social Support. Response option is categorized into ‘Yes’ or ‘No’.

**Table 3:** Adjusted model examining maternal psychosocial factors and characteristics on self report of exclusive breastfeeding

		Adjusted model	
Fixed effect		Odds ratio (95% CI)	*p*
Perceived stress^a^		0.88 (0.83–0.92)	<0.001
Received support^b^		1.48 (1.04–2.11)	0.032
Perceived social support^c^		2.54 (0.50–13.10)	0.263
Race			
White		Ref	Ref
Black		0.16 (0.02–1.40)	0.098
Education			
High school diploma/GED or less		Ref	Ref
College degree or higher		0.94 (0.06–13.11)	0.962
Employment status			
Employed		Ref	
Unemployed		0.58 (0.09–3.90)	0.578
Maternal age		0.96 (0.77–1.16)	0.642
**Random effects**	**Estimate (95% CI)**		
Variance	5.67 (4.99–6.43)	4.65 (3.89–5.57)	
PCV^d^ (%)		5.49	
ICC	0.91 (0.88–0.93)	0.86 (0.82–0.90)	
**Maximum likelihood estimate**			
AIC		3660.86	
BIC		3721.89	

Abbreviations: AIC, Akaike's Information Criterion; BIC, Bayesian Information Criterion; CI, confidence interval; ICC, Intraclass correlation; PCV, proportional change in variance.

^a^ Perceived Stress: Adapted Cohen's Perceived Stress Scale. Operationalized as a continuous variable.

^b^ Received support (Breastfeeding support): Is there a person/group/organization (e.g., family, professionals) that is helping you or providing any support (e.g., resources, emotional) to continue to breastfeed? Response option is ‘Yes’ or ‘No’.

^c^ Perceived social support: Adapted Multidimensional Scale of Perceived Social Support. Response option is categorized into ‘Yes’ or ‘No’.

^d^ PCV expresses the change in the area level variance between the empty model and the individual level model, and between the individual level model and the model further including the area level covariate.

### 4.4 Effect of race and EMA reports of stress on exclusive breastfeeding

Tables 4 and 5 show results from the mixed‐effects logistic regression examining the effect of stress, race, on exclusive breastfeeding. Adjusting for potential confounders in both Models 1 and 2, perceived stress is associated with decreased odds of exclusive breastfeeding. In Model 1, with an increase in perceived stress score, there was a 16% reduction in the odds of exclusive breastfeeding. Similarly, in Model 2, an increase in perceived stress score is associated with a 10% decrease in the odds of exclusive breastfeeding. Black participants were 99% less likely than White participants to breastfeed exclusively in Model 1 (OR: 0.01, 95% CI: 0.001–0.11) and 87% less likely to breastfeed exclusively compared with Whites in Model 2 (OR: 0.13, 95% CI: 0.02–0.78).

**Table 4:** Longitudinal mixed‐effect logistic model of exclusive breastfeeding, stress, perceived social support and race

	Intercept‐only model	Model 1 (Perceived social support as interaction term)	
	Odds ratio (95% CI)	Odds ratio (95% CI)	*p*
**Fixed effects**			
Perceived stress^a^		0.81 (0.76–0.87)	<0.001
Perceived social support^b^		0.89 (0.16–4.84)	0.889
Race			
White		Ref	Ref
Black		0.01 (0.001–0.072)	<0.001
Perceived social support*stress^c^		1.18 (1.07–1.30)	0.001
Perceived stress*race^d^		1.19 (1.04–1.35)	0.011
Education			
High school diploma/GED or less		Ref	Ref
College degree or higher		4.55 (0.44–47.79)	0.206
Employment status			
Employed		Ref	
Unemployed		0.11 (0.02–0.64)	0.014
Maternal age		1.00 (0.81–1.24)	0.982
**Random effects**	**Estimate (95% CI)**	**Estimate (95% CI)**	
Variance	5.67 (4.99–6.43)	5.41 (4.59–6.39)	
PCV^e^ (%)	Ref	4.59	
ICC	0.91 (0.88–0.93)	0.89 (0.86–0.92)	
**Maximum likelihood estimate**			
AIC	8793.79	4370.14	
BIC	8809.48	4441.24	

Abbreviations: AIC, Akaike's Information Criterion; BIC, Bayesian Information Criterion; CI, confidence interval; ICC, Intraclass correlation; PCV, proportional change in variance.

^a^ Perceived Stress: Adapted Cohen's Perceived Stress Scale. Operationalized as a continuous variable.

^b^ Perceived social support: Adapted Multidimensional Scale of perceived social support. Response option is categorized into ‘Yes’ or ‘No’.

^c^ Perceived social support*stress: Interaction term between perceived social support and perceived stress.

^d^ Perceived stress*race: Interaction term between perceived stress and race

^e^ PCV expresses the change in the area level variance between the empty model and the individual level model, and between the individual level model and the model further including the area level covariate.

**Table 5:** Longitudinal mixed‐effect logistic model of exclusive breastfeeding, stress, received social support (breastfeeding support) and race

	Intercept‐only model	Model 2 (Received social support as interaction term)	
	Odds ratio (95% CI)	Odds ratio (95% CI)	*p*
**Fixed effects**			
Perceived stress^a^		0.87 (0.82–0.93)	0.001
Received social support^b^ (Breastfeeding support)		2.26 (1.49–3.42)	<0.001
Race			
White		Ref	
Black		0.06 (0.01–0.40)	0.004
Received social support*stress^c^		0.96 (0.89–1.04)	0.323
Perceived stress*race^d^		1.26(1.11‐1.45)	0.001
Education			
High school diploma/GED or less		Ref	
College degree or higher		1.30 (0.13–13.2)	0.827
Employment status			
Employed		Ref	
Unemployed		0.37 (0.06–2.31)	0.289
Maternal age		0.99 (0.83–1.19)	0.924
**Random effects**	**Estimate (95% CI)**		
Variance	5.67 (4.99–6.43)	4.93 (4.19–5.81)	
PCV^e^ (%)		13.1	
ICC	0.91 (0.88–0.93)	0.88 (0.84–0.91)	
**Maximum likelihood estimate**			
AIC		4258.98	
BIC		4328.27	

Abbreviations: AIC, Akaike's Information Criterion; BIC, Bayesian Information Criterion; CI, confidence interval; ICC, Intraclass correlation; PCV, proportional change in variance.

^a^ Perceived Stress: Adapted Cohen's Perceived Stress Scale. Operationalized as a continuous variable.

^b^ Received support (Breastfeeding support): Is there a person/group/organization (e.g., family, professionals) that is helping you or providing any support (e.g., resources, emotional) to continue to breastfeed? Response option is ‘Yes’ or ‘No’.

^c^ Received support*stress: Interaction term between received social support and perceived stress.

^d^ Perceived stress*race: Interaction term between perceived stress and race.

^e^ PCV expresses the change in the area level variance between the empty model and the individual level model, and between the individual level model and the model further including the area level covariate.

### 4.5 Moderating effects of perceived social support and received social support (breastfeeding support) on stress

**Figure 1:**
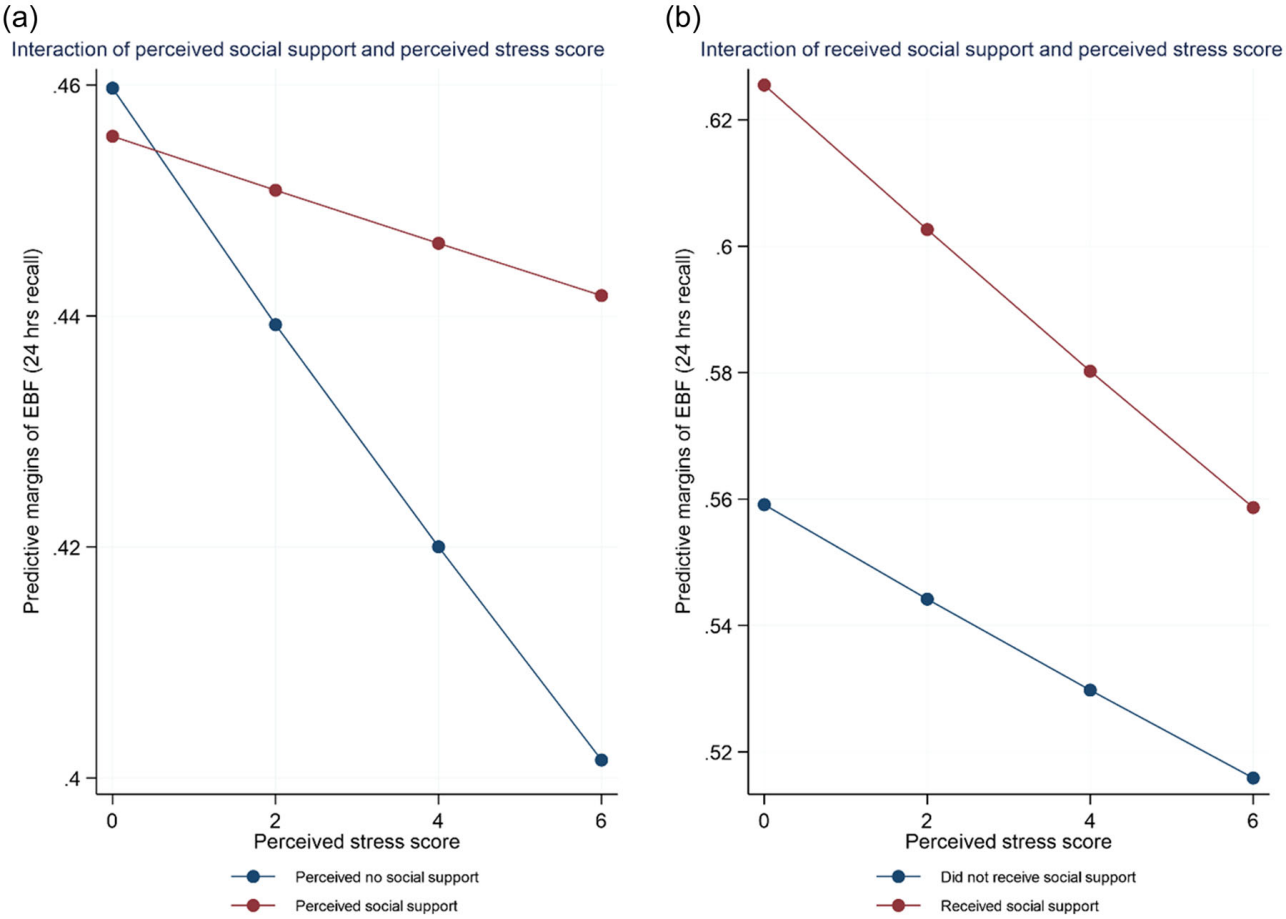
Interaction plots of perceived stress scores and exclusive breastfeeding. (a) Perceived social support. (b) Received social support.

Table 4 shows that perceived social support significantly moderated the relationship between perceived stress and exclusive breastfeeding (OR: 1.14, 95% CI: 1.04–1.26). Model 2 (Table 5) shows no significant moderating effect of received social support (breastfeeding support) on perceived stress (OR: 0.96, 95% CI: 0.89–1.04). Although there was no significant moderating effect of received social support on perceived stress, received social support had a significant association with exclusive breastfeeding (OR: 2.30, 95% CI: 1.52– 3.49). The interaction between perceived social support and stress is presented in Figure 1a. In Figure 1a, there was no remarkable difference in exclusive breastfeeding among individuals who perceived or did not perceive social support for participants with perceived stress score of zero (0). However, as the stress scores increased, those who perceived they had social support were more likely to report exclusive breastfeeding than those who did not perceive social support.

### 4.6 Interaction effect between race and stress

In Models 1 and 2, examining the interaction terms between stress and race, White participants with similar levels of stress like Black participants were less likely to exclusively breastfeed than Black participants. As stress scores increase, the odds of exclusively breastfeeding among Black participants compared with White participants increases.

### 4.7 Measures of variation

The null model (Model 0), Model 1 and Model 2 have high intraclass correlation (ICC) of 0.91, 0.89, and 0.88, respectively, implying good reliability in individual responses for exclusive breastfeeding. The observed ICC also indicates that the proportion of the total variance observed in our outcome, exclusive breastfeeding due to mean differences between subjects is high. We further calculated the proportional change in variance, which estimates the total variance attributable to the independent variables in the models. In the perceived social support model (Model 1), only 5% of the observed variation can be attributed to perceived stress, perceived social support and sociodemographic factors. In addition, 14% of the total variance observed in the received social support model (Model 2) was accounted for by received stress, perceived social support and sociodemographic factors.

## 5 DISCUSSION

The primary objective of this study was to examine the influence of perceived daily stress on exclusive breastfeeding. In addition to establishing the relationship between perceived stress and exclusive breastfeeding over 6 months, we sought to test whether perceived social support and received social (breastfeeding) support moderate this relationship. Furthermore, we tested the moderation effect of race on perceived stress. A key finding in the study is that participants who reported higher levels of perceived stress were less likely to breastfeed exclusively for 6 months. Our study results also show that the perception of social support moderated the relationship between perceived stress and breastfeeding. In contrast, received social (breastfeeding) support did not moderate the relationship between perceived stress and exclusive breastfeeding but directly increased the likelihood of exclusive breastfeeding. Black and unemployed birthing people were less likely to breastfeed exclusively within the study population. These findings have important implications for public health interventions to improve rates of exclusive breastfeeding. Results indicate that public health interventions to increase exclusive breastfeeding should consider parents’ support systems and life stressors.

Pregnancy and postpartum stages involve complex processes that may compound daily stress, making exclusive breastfeeding challenging. Despite methodological differences, our results corroborate studies that highlighted the influence of stress on exclusive breastfeeding (Dozier et al., 2012; Dugat et al., 2019). Although these studies operationalized stress as stressful life events rather than perceived stress, they demonstrated a significant relationship between stressful life events and decreased likelihood of exclusive breastfeeding. Others have cited possible reasons for the negative relationship between perceived stress and exclusive breastfeeding. Some researchers posit that elevated stress may induce hormonal responses that can reduce prolactin and oxytocin secretion, leading to inadequate milk supply or preventing the milk letdown reflex, thus causing exclusive breastfeeding cessation (Jalal et al., 2017; Mezzacappa et al., 2000; Mezzacappa, 2004; O'Brien et al., 2008). An alternative explanation from a recent prospective cohort study in Japan suggests that stress negatively affects exclusive breastfeeding through psychological burden and associated behavioral changes (Shiraishi et al., 2020). Exclusive breastfeeding can be labor‐intensive, at times fraught with negative emotions. It is possible that people experiencing stress from other things outside of breastfeeding may seek to decrease additional stress from exclusive breastfeeding. Our findings reinforce mounting evidence that perceived stress is significantly associated with nonexclusive breastfeeding.

In our study, participants who experienced higher stress levels but also perceived social support were more likely to breastfeed exclusively, compared with their counterparts without perceived social support. This finding is consistent with studies that have demonstrated the role of perceived social support from family, friends, and significant others in moderating the effect of stress on health behavior (Allgöwer et al., 2001; Brennan & Moos, 1992; Steptoe et al., 1996). Similarly, several studies have shown that partners, friends and family members provide psychological support, which may not necessarily be breastfeeding support needed to sustain exclusive breastfeeding (Bai et al., 2010; Bevan & Brown, 2014; Brand et al., 2011; Maleki‐Saghooni et al., 2020; Ogbo et al., 2020). This relationship can be linked to assertions that childbearing people who perceive they have social support from important persons in their lives adapt better to the postpartum period and cope with stress better in this period (Faridvand et al., 2017) all of which can lead to increased exclusive breastfeeding (Maleki‐Saghooni et al., 2020).

In contrast to the buffering role of perceived social support on participants with elevated perceived stress scores, receipt of breastfeeding support as reported by our study participants did not buffer their perceived stress. However, it directly increased the likelihood of exclusive breastfeeding among our study participants. This finding is similar to previous studies that showed that parents who received social support from health professionals, family, antenatal groups or postpartum breastfeeding support groups have increased breastfeeding self‐efficacy and, in turn, exclusively breastfed longer (Brown & Lee, 2011; Laugen et al., 2016; Mercan & Tari Selcuk, 2021). Breastfeeding is a learned behavior (Volk, 2009), and childbearing people can learn to breastfeed from lactation support providers. Learning to breastfeed can improve breastfeeding efficacy, which would increase exclusive breastfeeding. In addition, the receipt of breastfeeding support can directly solve practical problems that could lead to breastfeeding cessation (e.g., identification and treatment of mastitis, treatment for nipple pain/damage, etc.). As shown in our study, breastfeeding support such as support from health care professionals and lactation consultants may not necessarily buffer general stress, especially if the stress is not specific to the postpartum period and breastfeeding.

Childbearing people in our study were significantly less likely to exclusively breastfeed when compared with their White counterparts. Our finding confirms previous studies that have highlighted the racial gap and disparity in breastfeeding indices in the United States (Anstey et al., 2017; Beauregard et al., 2019; Jones et al., 2015). Black/African Americans have the lowest breastfeeding initiation rates, exclusive breastfeeding and breastfeeding duration than all other racial/ethnic groups. Researchers have also suggested that the long‐standing racial and ethnic differences in breastfeeding duration and exclusivity result from historical, cultural, social, economic, political, and psychosocial factors, including stress, which disproportionately affects Black birthing people (Jones et al., 2015; Louis‐Jacques et al., 2017; Troxel et al., 2003).

We hypothesized that Black participants who experienced high stress levels will less likely exclusively breastfeed than White participants who experienced similar high stress levels. However, our findings demonstrate that Black participants who experienced stress were more likely to exclusively breastfeed. In our study, the stress levels between White and Black participants were not significantly different. (see Supporting Information file) and as demonstrated in one of the study's publications (Omowale et al., 2021). This finding does not support other research studies that suggest that Black people may face an increased risk for stress, which impairs breastfeeding and contributes to the disparity observed in adverse birth outcomes (Giscombé & Lobel, 2005; Johnson et al., 2015b). This can be attributed to heightened stress levels experienced by the US public, especially during the early stages of the coronavirus disease 2019 (COVID‐19) pandemic, a nonnormative event for everyone regardless of race.

During the COVID‐19 pandemic, there were instances where stress levels among White people were unusually higher than that of Black people. For example, in a study conducted by the CDC, White adults were more likely to report stress and worry about the health of family members and loved ones than Black adults (McKnight‐Ely et al., 2021). It is important to note that many PMOMS’ participants contributed significant data to the study during the COVID‐19 pandemic as noted in one of PMOMS study publication (Omowale et al., 2021). The concept of coping and resiliency among Black people in the United States could have contributed to the observed different result from the norm where Black people who were stressed exclusively breastfed more than White people. Coping and resilience models suggest that some Black people in the United States have over time developed coping strategies and resilience that have enabled them to overcome difficulties, the negative consequences of their environments (Brown, 2008). Although Black people who experienced stress were more likely to exclusively breastfeed compared with White people, overall, in the study, Black people were less likely to exclusively breastfeed. This suggests that factors influencing breastfeeding are multifaceted and the need to develop breastfeeding interventions that address these nuances.

Our study has several strengths and limitations. One of the strengths is the use of EMA, a novel data collection method that samples subject experiences in real‐time, minimizing recall bias. The observed variation in stress and exclusive breastfeeding allowed us to examine the effect of daily reports of perceived stress and exclusive breastfeeding. Another strength is the use of a standardized scale to measure perceived stress and perceived social support. Previous studies that examined the relationship between stress and exclusive breastfeeding operationalized stress as stressful life events (Dozier et al., 2012; Dugat et al., 2019). However, not all stressful events can impact exclusive breastfeeding (Dozier et al., 2012).

A limitation of the study is our use of a single question to measure receipt of breastfeeding support rather than a multi‐item scale. This was a single question via an EMA survey, which is intended to be short and captured momentary experiences where longer surveys and scales captured multiple aspects of a phenomenon usually at one point in time. PSS and MSPSS used for the study were adapted or abridged versions of the original validated scales. However, the reliability test of the adapted PSS and MSPSS showed high internal consistency of Cronbach's *α* 0.81 and 0.95, respectively. This was also necessary to reduce respondent burden and fatigue.

Another limitation is that the MSPSS was administered only once during the study as the participant is exiting the survey, unlike other variables measured repeatedly to reduce respondent burden. We believe, however, that the perception of social support should not fluctuate substantially during this time period. A longitudinal validity (test–retest) of MSPSS scale conducted in a study provides evidence of the scale's stability over time (Dambi et al., 2018; Saeieh et al., 2017). In a study among postpartum mothers, perceived social support was stable for a period of 15 weeks (Saeieh et al., 2017). We also note that our sample was drawn from one county served by one maternity hospital in Pennsylvania, and as such, our findings may not be generalizable to other settings.

## 6 IMPLICATION FOR PRACTICE

To our knowledge, this study is the first longitudinal study to examine how different forms of social support and perceived stress influence exclusive breastfeeding. Our study suggests that perceived stress, perceived social support and receipt of breastfeeding support are important drivers of exclusive breastfeeding. Structural issues including unpaid maternity leave and racism are major sources of stress among birthing people (Troxel et al., 2003) and it is necessary to address these issues among birthing people. In many cases, stress could likely be reduced if parents had extended paid leave, easy access to breastfeeding support in hospital and after discharge, and access to racially concordant care from birth workers (Duncan et al., 2022; Lauzon‐Guillain et al., 2019). In addition, maternal stress reduction interventions such as prevention of breastfeeding complications, prenatal lactation counseling, preparation, especially among new parents and relaxation therapy to induce milk production during breastfeeding are effective interventions that can be explored (Shukri et al., 2018).

Our findings substantiate the need for breastfeeding interventions that can improve social support and maximize the use of birthing people's existing social networks. One potential intervention that can be scaled up is the use of racially diverse Community Health Workers (CHWs) as breastfeeding peer counselors providing culturally and linguistically appropriate breastfeeding counseling and support services. The engagement of these CHWs in home visiting programs is a proven way to promote and support breastfeeding (Chapman et al., 2010). CHWs are uniquely positioned to address racial health disparities that disproportionately affect communities of color. Expanding their scope to provide breastfeeding services can help reduce structural barriers to breastfeeding such as access to information and services Black birthing people face.

In addition, the scope of work for Doulas can be expanded to provide breastfeeding support services. We also recommend that public health program implementers design breastfeeding support programs involving partners, family and friends, as these individuals play an important role in buffering stress, especially among minority groups. Further studies are needed to determine the potential sources of stress among breastfeeding people and how different types of social support, such as emotional, informational, appraisal and tangible support, will affect exclusive breastfeeding.

## AUTHOR CONTRIBUTIONS

Chinwoke Isiguzo, Dara D. Mendez, Jill R. Demirci, Ada Youk, and Patricia Documet conceptualized the study. Dara D. Mendez, Esa M. Davis, and Gabriella Mendez designed the parent study. Chinwoke Isiguzo analyzed the data. Chinwoke Isiguzo wrote the original draft. Chinwoke Isiguzo, Dara D. Mendez, Jill R. Demirci, Ada Youk, Gabriella Mendez, Esa M. Davis, and Patricia Documet reviewed and edited the original draft. Dara D, Mendez, Jill R. Demirci, Ada Youk, and Patricia Documet supervised the study. Dara D. Mendez was in charge of funding acquisition.

## CONFLICT OF INTEREST

Esa M. Davis is a member of the United States Preventive Services Task Force (USPSTF). This article does not necessarily represent the views and policies of the USPSTF.

## ETHICS STATEMENT

The Human Research Protection Office at the University of Pittsburgh approved this study protocol in October 2017 under #PRO16100117.

## Supporting information

Supporting information.

## Data Availability

The data that support the findings of this study are available from the corresponding author upon reasonable request.
