# Supplementary material for: Stress, social support, and racial differences: Dominant drivers of exclusive breastfeeding
Source: Matern Child Nutr. 2022 Nov 21;19(2):e13459. doi: 10.1111/mcn.13459 (PMC10019056; doi:10.1111/mcn.13459)
Supplement: Supplementary file 1 — Supporting information. [file MCN-19-e13459-s001.docx]

**Supplementary File**

| **Table S1: Mean stress levels stratified by race** | | |  |  |
| --- | --- | --- | --- | --- |
| Race | Mean Stress Levels | Standard Deviation | Minimum | Maximum |
| All | 3.30 | 2.59 | 0 | 13.85 |
| White | 3.37 | 2.60 | 0 | 13.85 |
| Black | 3.14 | 2.60 | 0 | 9.07 |
